# Supplementary material for: Electrochemical Investigation of PEDOT:PSS/Graphene Aging in Artificial Sweat
Source: Polymers (Basel). 2024 Jun 14;16(12):1706. doi: 10.3390/polym16121706 (PMC11207453; doi:10.3390/polym16121706)
Supplement: Supplementary file 1 [file polymers-16-01706-s001.zip › polymers-3029463-supplementary.pdf]

Supplementary Materials

Electrochemical investigation of PEDOT:PSS/Graphene aging in artificial sweat

Boriana Tzaneva [1,*], Valentin Mateev [2], Bozhidar Stefanov [1], Mariya Aleksandrova [3] and Ivo Iliev [4]

[1] Department of Chemistry, Faculty of Electrical Engineering and Technology, Technical University of Sofia, Kliment Ohridski Blvd., 8, 1000 Sofia, Bulgaria; borianatz@tu-sofia.bg (B.T.); b.stefanov@tu-sofia.bg (B.S.)
[2] Department of Electrical Apparatus, Faculty of Electronic Engineering, Technical University of Sofia, Kliment Ohridski Blvd., 8, 1000 Sofia, Bulgaria; vmateev@tu-sofia.bg
[3] Department of Microelectronics, Faculty of Electronic Engineering and Technology, Technical University of Sofia, Kliment Ohridski Blvd., 8, 1000 Sofia, Bulgaria; m_aleksandrova@tu-sofia.bg
[4] Department of Electronics, Faculty of Electronic Engineering and Technology, Technical University of Sofia, Kliment Ohridski Blvd., 8, 1000 Sofia, Bulgaria
* Correspondence: borianatz@tu-sofia.bg

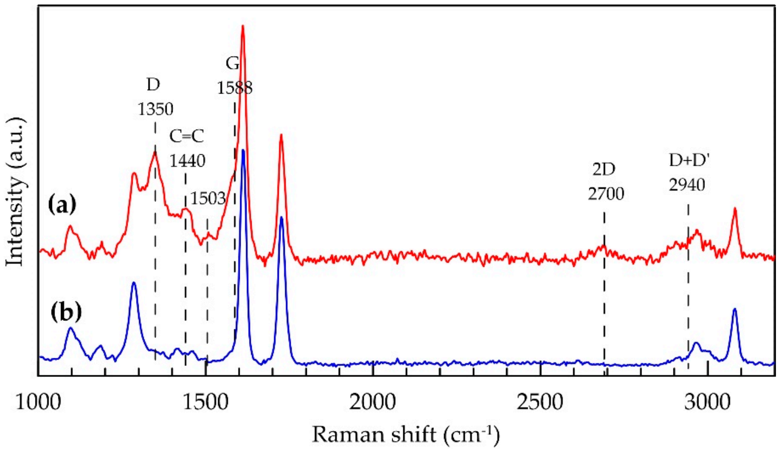

Figure S1. Raman spectra of PEDOT:PSS/Graphene ink. (a) PEDOT:PSS/Graphene dropped on PET; (b) PEDOT:PSS/Graphene sprayed on PET.

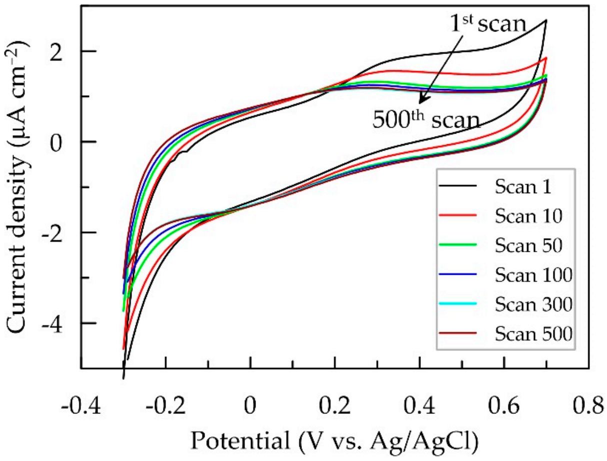

Figure S2. Progressive shape change in CV plot of the PEDOT:PSS/Graphene layer in artificial sweat.

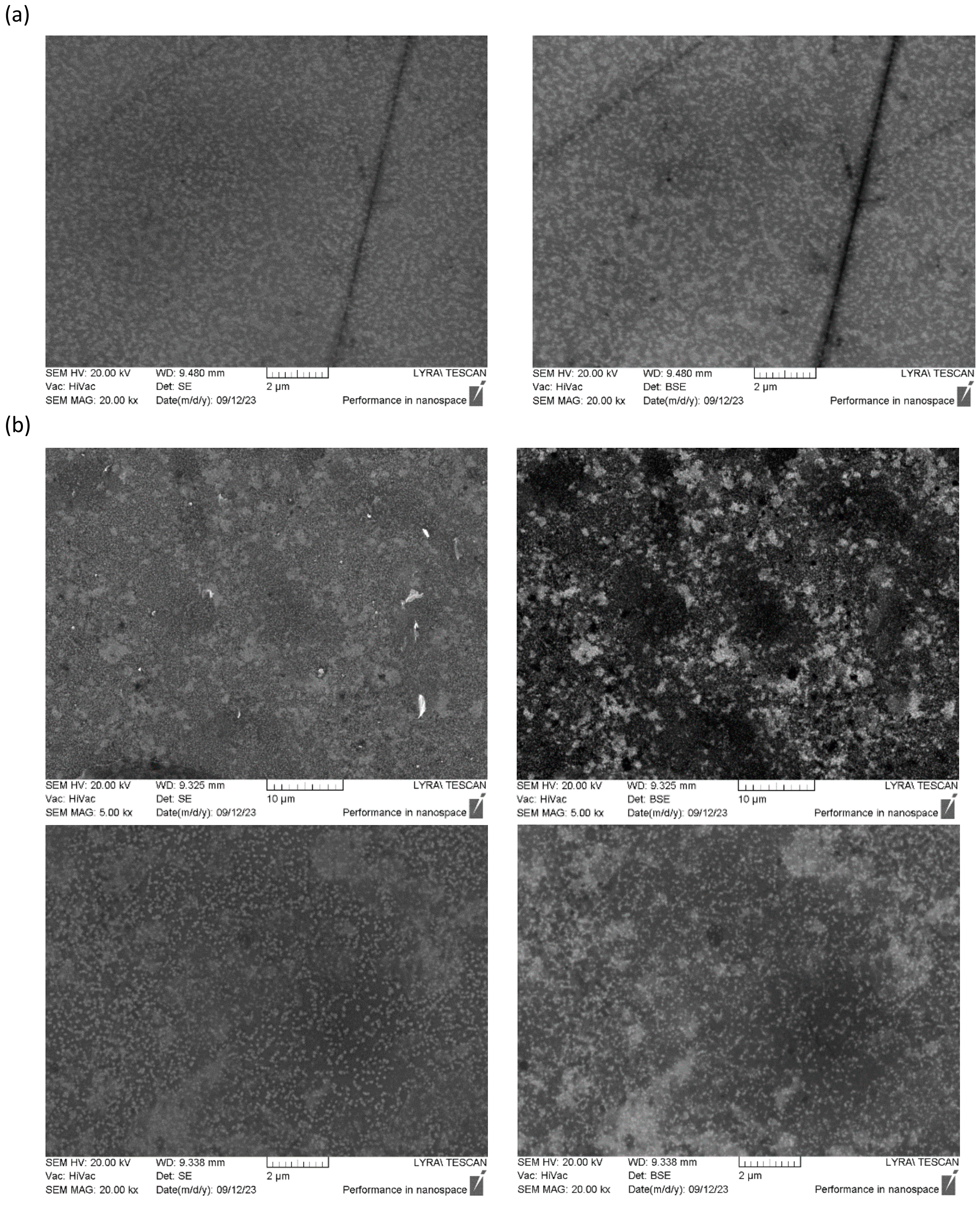

Figure S3. SEM raw imagines of PEDOT:PSS/Graphene layer. (a) before electrochemical testing in SE and BES mode; (b) after 500 scans in artificial sweat in SE (in left) and BES (in right) mode in low and high magnification.

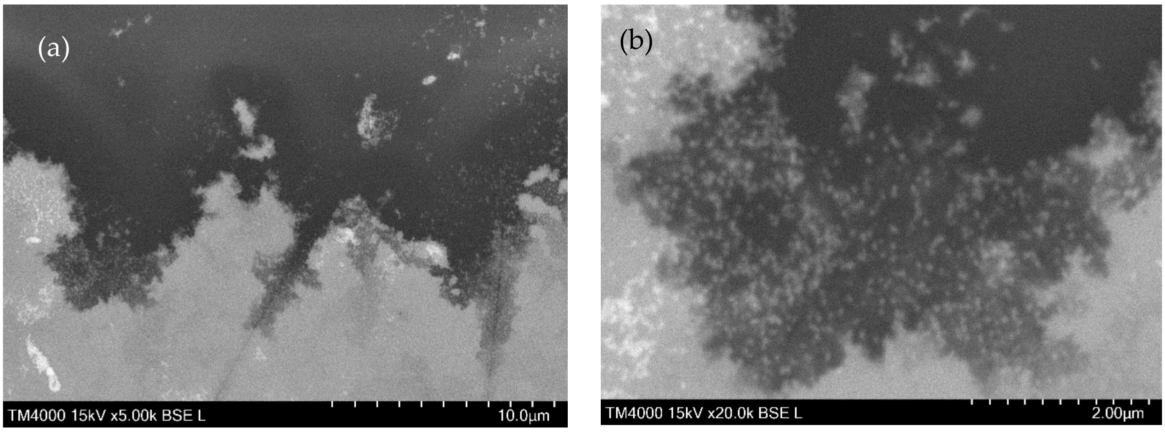

Figure S4. SEM imagines in BSE mode of the PEDOT:PSS/Graphene layer after 9 days in artificial sweat and polarization tests at low magnification (a) and at high magnification (b).

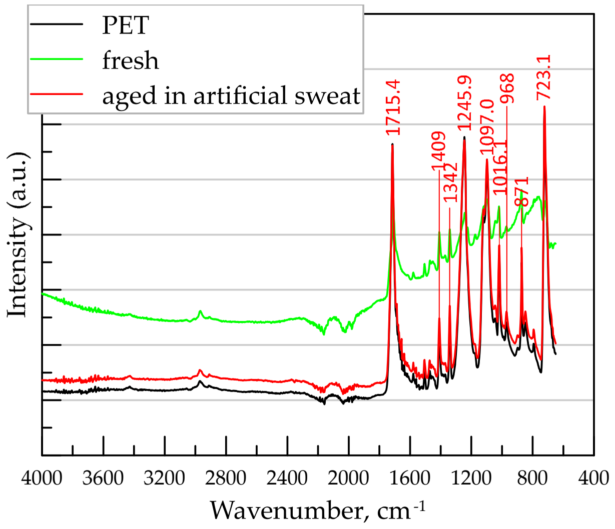

Figure S5. FTIR spectra of PET substrate, fresh and aged in artificial sweat PEDOT:PSS/Graphene sprayed on PET.
